# Structural dynamics of double-stranded DNA with epigenome modification

**DOI:** 10.1093/nar/gkaa1210

**Published:** 2020-12-18

**Authors:** Ayako Furukawa, Erik Walinda, Kyohei Arita, Kenji Sugase

**Affiliations:** Graduate School of Medical Life Science, Yokohama City University, 1-7-29 Suehiro-cho, Tsurumi-ku, Yokohama, Kanagawa 230-0045, Japan; Bioorganic Research Institute, Suntory Foundation for Life Sciences, 8-1-1 Seikadai, Seika, Soraku, Kyoto 619-0284, Japan; Department of Molecular and Cellular Physiology, Graduate School of Medicine, Kyoto University, Yoshida Konoe-cho, Sakyo-ku, Kyoto 606-8501, Japan; Graduate School of Medical Life Science, Yokohama City University, 1-7-29 Suehiro-cho, Tsurumi-ku, Yokohama, Kanagawa 230-0045, Japan; Bioorganic Research Institute, Suntory Foundation for Life Sciences, 8-1-1 Seikadai, Seika, Soraku, Kyoto 619-0284, Japan; Department of Molecular Engineering, Graduate School of Engineering, Kyoto University, Kyoto-Daigaku Katsura, Nishikyo-Ku, Kyoto 615-8510, Japan

## Abstract

Modification of cytosine plays an important role in epigenetic regulation of gene expression and genome stability. Cytosine is converted to 5-methylcytosine (5mC) by DNA methyltransferase; in turn, 5mC may be oxidized to 5-hydroxymethylcytosine (5hmC) by ten-eleven translocation enzyme. The structural flexibility of DNA is known to affect the binding of proteins to methylated DNA. Here, we have carried out a semi-quantitative analysis of the dynamics of double-stranded DNA (dsDNA) containing various epigenetic modifications by combining data from imino ^1^H exchange and imino ^1^H *R*_1ρ_ relaxation dispersion NMR experiments in a complementary way. Using this approach, we characterized the base-opening (*k*_open_) and base-closing (*k*_close_) rates, facilitating a comparison of the base-opening and -closing process of dsDNA containing cytosine in different states of epigenetic modification. A particularly striking result is the increase in the *k*_open_ rate of hemi-methylated dsDNA 5mC/C relative to unmodified or fully methylated dsDNA, indicating that the Watson–Crick base pairs undergo selective destabilization in 5mC/C. Collectively, our findings imply that the epigenetic modulation of cytosine dynamics in dsDNA mediates destabilization of the GC Watson–Crick base pair to allow base-flipping in living cells.

## INTRODUCTION

Cytosine modifications are known to play an important role in epigenetic regulation of gene expression and genome stability (1,2). Cytosine is converted to 5-methylcytosine (5mC) by DNA methyltransferases (3,4); in turn, 5mC may be further oxidized to 5-hydroxymethylcytosine (5hmC) by ten-eleven translocation (TET) enzyme (5,6). Many proteins specifically recognize these DNA modifications. For example, UHRF1 (ubiquitin-like containing PHD and RING finger domains 1) specifically recognizes hemi-methylated double-stranded DNA (dsDNA), in which one strand is methylated but the other is not. UHRF1 binds specifically to the ‘flipped-out’ 5mC structure (7–9), but does not interact with either unmodified dsDNA or full-methylated dsDNA, in which both strands are methylated (7). Although crystal structures of UHRF1 in the free and 5mC–DNA-bound forms have been reported (7–9), the mechanism by which the 5mC nucleotide flips out from the inside of the dsDNA for specific recognition by UHRF1 remains elusive.

One possibility is that DNA methylation increases the flexibility of dsDNA, thereby enabling the dsDNA at the position of 5mC to more frequently adopt a flipped-out structure or at least to adopt a more open structure that can then be recognized by UHRF1. Indeed, it has been shown that DNA methylation can alter DNA flexibility and affect the properties of proteins binding to the methylated DNA (10–12). Even without methylation, dsDNA is not completely rigid, but rather has a degree of flexibility. A particularly intriguing example of this flexibility is that the hydrogen bonds that form a Watson–Crick (WC) base pair in canonical dsDNA can be transiently broken, enabling the WC base pair to interconvert to a Hoogsteen (HG) base pair (13). The hydrogen bond break permits ^1^H exchange (which can be experimentally observed) between water and the imino proton in thymine and guanine, even if the WC base pair is simply broken and does not fully interconvert into an HG base pair.

The ^1^H exchange rate (*k*_1H_) of the imino proton has been measured for various dsDNA constructs by using ^1^H NMR. In this type of experiment, a catalyst, typically ammonium, is often used to enhance the *k*_1H_ rate and to estimate the equilibrium constant *K*_op_, which is the ratio of the base-opening rate (*k*_open_) to the base-closing rate (*k*_close_). However, ammonium can induce conformational changes in dsDNA and change the ^1^H spin relaxation rate, thereby altering the derived *k*_1H_ rate (14,15). In the case of 5hmC, for example, a hydrogen bond is formed between its hydroxyl group and the next base via water molecules, which may be disturbed by catalysts (16). Therefore, although it is difficult to estimate *K*_op_ without catalysts, their use should be avoided in the comparison of *k*_1H_ rates among different dsDNA samples, such as those with cytosine bases in different states of epigenetic modification.

^1^H exchange NMR experiments can indirectly characterize the base-opening and -closing process by permitting derivation of the equilibrium constant *K*_op_. The base-opening and -closing process is usually a very fast process occurring on the microsecond timescale; therefore, it is very hard to probe under the conditions of physiological pH, temperature, and buffer, where the lifetime of the open state is very short-lived and its population is extremely low. By contrast, NMR relaxation dispersion experiments can provide the kinetic exchange rate (*k*_ex_), which is the sum of *k*_open_ and *k*_close_, even in the case where the population of the open state is too small to be directly observed by NMR (17,18). Indeed, the development of ^13^C and ^15^N *R*_1ρ_ NMR relaxation dispersion has facilitated a direct characterization of the base-opening and -closing process, and even the above-mentioned WC to HG base pair exchange process (13,19–22).

In this study, we have carried out a semi-quantitative analysis of the base-opening and -closing process of unmodified and modified dsDNA samples by combining ^1^H NMR *R*_1ρ_ relaxation dispersion with ^1^H exchange NMR to obtain insight into the mechanism by which the base-opening process may be exploited for the selective recognition of epigenetically modified dsDNA by proteins such as UHRF1. This combined NMR method can be applied without catalysts to study dsDNA dynamics even at neutral pH, provided that the base-closing rate is much faster than the intrinsic ^1^H exchange rate, a condition that is commonly fulfilled for ^1^H exchange NMR experiments of dsDNA (14–16). Using this method, we report a site-specific comparison of the base-opening and -closing process in dsDNA containing cytosine in different epigenetic modification states.

## MATERIALS AND METHODS

### Oligonucleotides

**Figure 1.**
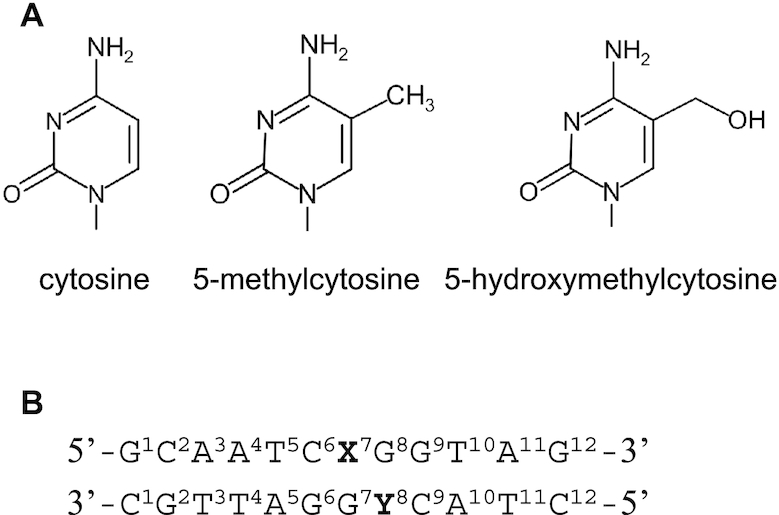
Structures of cytosine, the modified cytosine bases and the double-stranded DNA (dsDNA) sequence used. (**A**) Chemical structures of cytosine (**C**), 5-methylcytosine (5mC), and 5-hydroxymethylcytosine (5hmC). (**B**) The dsDNA sequence with the numbering used in this study. X and Y are C, 5mC or 5hmC.

DNA oligonucleotides, synthesized and purified by high-performance liquid chromatography, were purchased from FASMAC (Figure 1 and Table 1). To prepare the experimental dsDNA samples, each oligonucleotide was suspended in 150 mM NaCl solution (pH 7.5 adjusted by NaOH), heated at 95°C for 5 min, and annealed by gradual cooling to 25°C.

**Table 1.** Melting temperature, *T*_m_, of the six dsDNA samples

Sample	*T*_m_ (°C)
C/C	55.37 ± 0.09
5mC/C	55.43 ± 0.21
5mC/5mC	56.37 ± 0.28
C/5mC	56.42 ± 0.27
5hmC/C	55.04 ± 0.11
5hmC/5mC	56.00 ± 0.43

### Thermal melting experiments

The melting temperature of 2 μM dsDNA samples was determined by using a V-650 UV-VIS spectrophotometer (JASCO). The samples were heated over a temperature range of 25–95°C at a rate of 1°C min^−1^. The melting temperature (*T*_m_) of each sample was derived from the first-order derivative of the melting curve.

### NMR experiments

The dsDNA samples were dissolved at a final concentration of 300 μM in 150 mM NaCl solution (pH 7.5) containing 5% D_2_O. All NMR experiments were carried out on an AVANCE DRX 600 spectrometer equipped with a TXI cryogenic probe and z-axis gradient or an AVANCE III HD 600 spectrometer equipped with a TCI cryogenic probe and z-axis gradient (Bruker). A 2D ^1^H nuclear Overhauser spectroscopy (NOESY) spectrum was measured for each dsDNA at 15°C to obtain sequential NOE connectivities between imino ^1^H resonances. The NMR spectra were processed by using NMRPipe (23), and imino ^1^H resonances were assigned on the basis of NOE connectivities. The resultant assignments were transferred to those of the resonances at 30°C by measuring a series of one-dimensional (1D) NMR spectra at temperatures from 15 to 30°C. The imino ^1^H resonances of G1, A11 and G12, which are located at or near either end of the dsDNA, remained unassigned because their flexibility provided no NOE connectivities at 15°C.

Water–imino proton exchange rates, *k*_1H_, were estimated from 1D CLEANEX-PM experiments at 30°C with mixing times *τ*_m_ of 5, 10, 15, 20, 50, 100 and 200 ms (24). Reference spectra were measured by the same pulse program with the CLEANEX-PM block omitted. NMR signals were deconvoluted if overlapping, and signal intensities were obtained by using TopSpin (Bruker). The obtained CLEANEX-PM intensities were fitted to the following equation by using the program GLOVE (25):

(1) $$\begin{eqnarray*}\frac{{I\left( {{\tau _{\rm{m}}}} \right)}}{{{I_0}}} &=& \frac{{{k_{1{\rm{H}}}}}}{{{R_{\rm{A}}} + {k_{1{\rm{H}}}} - {R_{\rm{B}}}}}\nonumber\\ &&\times\left\{ {{\rm{exp}}\left( { - {R_{\rm{B}}}{\tau _{\rm{m}}}} \right) - {\rm{exp}}\left[ { - \left( {{R_{\rm{A}}} + {k_{1{\rm{H}}}}} \right){\tau _{\rm{m}}}} \right]} \right\},\end{eqnarray*}$$

where *I*(*τ*_m_) and *I*_0_ are the signal intensities measured at *τ*_m_ and measured as a reference, respectively; and *R*_A_ and *R*_B_ are the apparent relaxation rates of the imino proton and the water proton, respectively.

**Figure 2.**
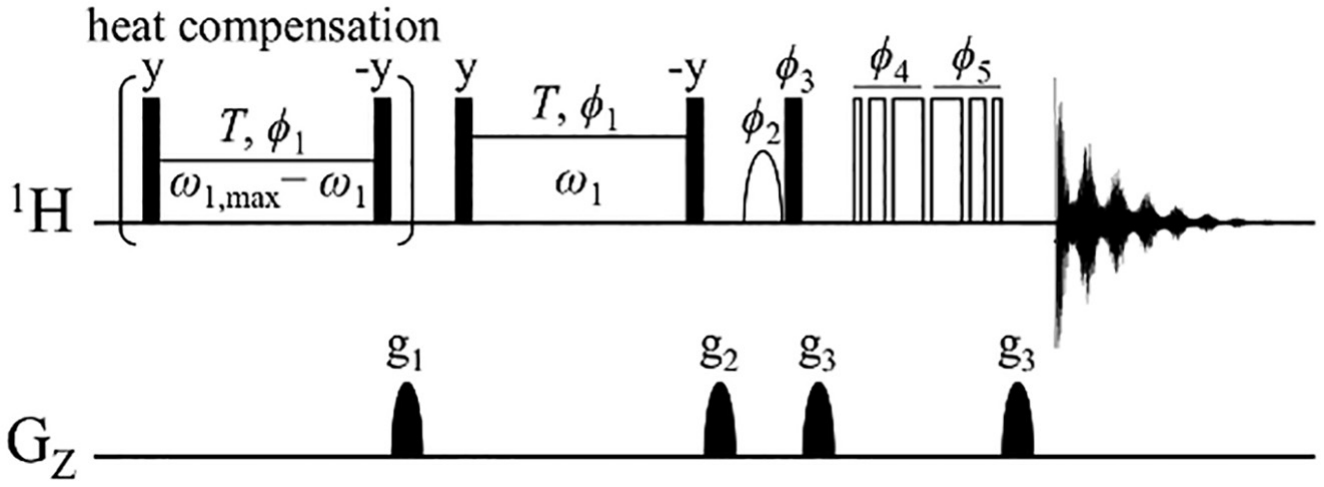
Pulse scheme for on-resonance imino proton *R*_1ρ_ dispersion in dsDNA. All ^1^H 90° radio-frequency (RF) pulses are shown as filled bars. The shaped 90° ^1^H pulse shown as a half ellipse is the water-selective 1-ms sinc pulse. The heat compensation block is applied at 300 ppm downfield to maintain the sample at a constant temperature throughout the measurements. The RF power of the heat compensation pulse is varied as *ω*_1,max_ – *ω*_1_, where *ω*_1_,_max_ is the maximum spin-lock power *ω*_1_ used in a set of *R*_1ρ_ dispersion measurements. After the g_1_ gradient that eliminates initial unwanted transverse magnetization, the carrier frequency is jumped to an imino ^1^H resonance of interest. The on-resonance ^1^H magnetization is rotated by 90° and spin-locked at a spin-lock field strength *ω*_1_ with a constant length *T*. The ^1^H magnetization is flipped back to the z-axis, followed by a z-filter gradient g_2_. The 3–9–19 WATERGATE pulses are applied at the carrier frequency of 4.7 ppm to suppress the water signal, and lastly the 1D ^1^H NMR spectrum is acquired. The phase cycle is given as follows: *ϕ*1 = (*x*); *ϕ*2 = (–*x*, *x*); *ϕ*3 = (*x*, –*x*); *ϕ*4 = (*x*, *x*, *y*, *y*, –*x*, –*x*, –*y*, –*y*); *ϕ*5 = (–*x*, –*x*, –*y*, –*y*, *x*, *x*, *y*, *y*); rec = (*x*, –*x*, –*x*, *x*).

1D on-resonance imino ^1^H *R*_1ρ_ relaxation dispersion experiments were carried out at 30°C by using the pulse sequence shown in Figure 2. A similar pulse sequence in which the carrier frequency was set in the middle of the imino proton spectral region was recently reported (26). In our case, we set the carrier frequency on-resonance to each imino proton resonance. An advantage of the ^1^H *R*_1ρ_ relaxation dispersion experiment is that higher radio-frequency power can be used for ^1^H than for other nuclei because of its higher gyromagnetic ratio, which means that ^1^H *R*_1ρ_ relaxation dispersion can detect faster conformational exchange as compared with its ^13^C and ^15^N counterparts. Furthermore, the ^1^H *R*_1ρ_ relaxation dispersion experiment does not require isotope-labeled samples. It is possible that the derived ^1^H *R*_1ρ_ relaxation rates may be influenced by ^1^H–^1^H cross-relaxation. Therefore, we theoretically estimated the influence of this cross-relaxation on the ^1^H *R*_1ρ_ relaxation dispersion measurement (Supplementary Methods, Supplementary Figures S1 and S2, Supplementary Tables S1 and S2), which showed that ^1^H–^1^H cross-relaxation is negligible for the conditions (described below) under which the ^1^H *R*_1ρ_ relaxation dispersion profiles of the dsDNA samples were obtained.

^1^H *R*_1ρ_ relaxation spectra were measured at spin-lock powers, *ω*_1_/(2π), ranging from 0.5 to 15 kHz with a constant relaxation period *T* of 20 ms. A reference spectrum was measured with the pulse sequence that omitted the spin-lock period. All *R*_1ρ_ dispersion spectra were Fourier-transformed, baseline-corrected, and peak-integrated by using TopSpin. The effective *R*_1ρ_ relaxation rate was calculated as:

(2) $$\begin{equation*}{R_{1\rho }} = - \frac{1}{T}{\rm{ln}}\left( {\frac{{{I_{{\rm{SL}}}}}}{{{I_0}}}} \right),\end{equation*}$$

where *I*_SL_ and *I*_0_ are the signal intensities of the imino proton resonances of interest measured in the presence and absence of the spin-lock, respectively. Errors in the effective *R*_1ρ_ relaxation rates were derived from the amplitude of the baseline noise. The resulting *R*_1ρ_ dispersion profiles were fitted to the following equation for a two-state exchange process (A ↔ B) by using GLOVE (25):

(3) $$\begin{eqnarray*}{R_{1\rho }} = R_2^0 + \frac{{{p_{\rm{A}}}{p_{\rm{B}}}\Delta {\omega ^2}{k_{{\rm{ex}}}}}}{{\frac{{\omega _{{\rm{Ae}}}^2\omega _{{\rm{Be}}}^2}}{{\omega _{\rm{e}}^2}} + k_{{\rm{ex}}}^2 - {p_{\rm{A}}}{p_{\rm{B}}}\Delta {\omega ^2}\left( {1 + \frac{{2k_{{\rm{ex}}}^2\left( {{p_{\rm{A}}}\omega _{{\rm{Ae}}}^2 + {p_{\rm{B}}}\omega _{{\rm{Be}}}^2} \right)}}{{\omega _{{\rm{Ae}}}^2\omega _{{\rm{Be}}}^2 + \omega _{\rm{e}}^2k_{{\rm{ex}}}^2}}} \right)}}\nonumber\\ \end{eqnarray*}$$

$$\begin{equation*}\omega _{{\rm{Ae}}}^2 = \omega _1^2\end{equation*}$$

$$\begin{equation*}\omega _{{\rm{Be}}}^2 = \Delta {\omega ^2} + \omega _1^2\end{equation*}$$

$$\begin{equation*}\omega _{\rm{e}}^2 = {\rm{\Omega }}_{\rm{A}}^2 + \omega _1^2\end{equation*}$$

$$\begin{equation*}{{\rm{\Omega }}_{\rm{A}}} = - \frac{{{p_{\rm{B}}}k_{{\rm{ex}}}^2{\rm{\Delta }}\omega }}{{k_{{\rm{ex}}}^2 + \Delta {\omega ^2}}},\end{equation*}$$

where *R*_2_^0^ is the intrinsic transverse relaxation rate (18). *p*_A_ and *p*_B_ (*p*_A_ + *p*_B_ = 1) are the populations of the major and minor states, respectively. *k*_ex_ denotes the chemical exchange rate (i.e. *k*_open_ + *k*_close_). Δ*ω* is the chemical shift difference between the two states. Note that, for fast exchange on the chemical shift timescale (*k*_ex_ >> Δ*ω*), it is impossible to separate *p*_A_ and *p*_B_ from *p*_A_*p*_B_ Δ*ω*^2^ by this method.

### Molecular dynamics

All molecular dynamics simulations were performed in explicit water using GROMACS version 5.1 (27–31). The Amber99sb force field with the ParmBSC0 nucleic acid parameters was used (32). The starting structures were ideal DNA double helices obtained from the program 3DNA (33,34) using the sequence given in Table 1. Force field parameters for 5mC were obtained from ref. (35). Force field parameters for 5hmC were constructed as described previously (36). The respective DNA was solvated in a triclinic box with SPC/E water. The temperature and salt concentration in the simulations were chosen to mimic the experimental conditions: that is, the system charge was neutralized by addition of sodium (Na^+^) and chloride (Cl^-^) ions to a total salt concentration of 150 mM. After solvation, energy minimization was carried out by the steepest-descent algorithm (maximum force: 1000 kJ mol^−1^ nm^−1^). The system was equilibrated as follows: first, the system was simulated under the canonical (NVT) ensemble for a time of 100 ps; and second, 100 ps of NPT simulation was performed. In both steps, position restraints were applied on all DNA atoms. Pressure coupling was achieved by the Parrinello–Rahman algorithm (37) (1 bar, 2.0-ps coupling constant). Temperature coupling was achieved by the modified Berendsen (29) thermostat (303 K, 0.1-ps coupling constant). The integration time step was set to 2 fs. Bonds were constrained by the LINCS algorithm (31). The parameters for fast particle mesh Ewald electrostatics (38) were as follows: Coulomb cut-off distance, 0.8 nm; fast Fourier transform grid spacing, 0.08 nm; Lennard–Jones cut-off distance, 0.8 nm. The interpolation order was cubic. Initial velocities were assigned from the Maxwell distribution at 303 K. Production molecular dynamics (MD) ran for a total duration of 1 μs. All calculations were performed on Linux workstations using CUDA-compatible NVIDIA graphics processing units (∼50 ns/day). Processing of the trajectories was carried out by tools integrated in GROMACS and 3DNA. Distributions were analysed by numpy and scipy using matplotlib for visualization (39–41).

## RESULTS

### Specific design of epigenetically distinct dsDNA constructs

We designed six dsDNA sequences using the deoxyribonucleic acids carrying 5mC and 5hmC modifications based on the sequence recognized by UHRF1 (7). The samples were designated as unmodified dsDNA (C/C), hemi-methylated dsDNA at nucleotide position 7 (5mC/C), full-methylated dsDNA (5mC/5mC), hemi-methylated dsDNA at nucleotide position 8 (C/5mC), hemi-hydroxymethylated dsDNA (5hmC/C), and hydroxymethyl-methylated dsDNA (5hmC/5mC) (Figure 1). These dsDNA sequences were identical except for the state of cytosine modification at nucleotide positions 7 and 8. Because 5mC/C is recognized by UHRF1, it is the most physiologically relevant sequence, as highlighted by the crystal structure of the UHRF1–dsDNA complex (7–9). C/C and 5hmC/C were control samples, each differing from 5mC/C at a single (un)modified base, to analyse the effects of epigenetic cytosine modification on dsDNA dynamics. C/5mC, 5mC/5mC and 5hmC/5mC were also control samples designed to examine the effect of methylation at nucleotide position 8.

### Epigenetic cytosine modification does not affect the overall thermal stability of dsDNA

To examine whether the overall thermal stability of dsDNA differs depending on the type of cytosine modification, we measured the DNA melting temperature (*T*_m_) of the six dsDNA samples by using a UV/Vis spectrometer. Interestingly, the *T*_m_ values of all the dsDNA samples were almost identical (Table 1), indicating that site-specific cytosine modification does not significantly change the overall stability of dsDNA.

### Cytosine modification alters the chemical environment of the guanine imino proton

**Figure 3.**
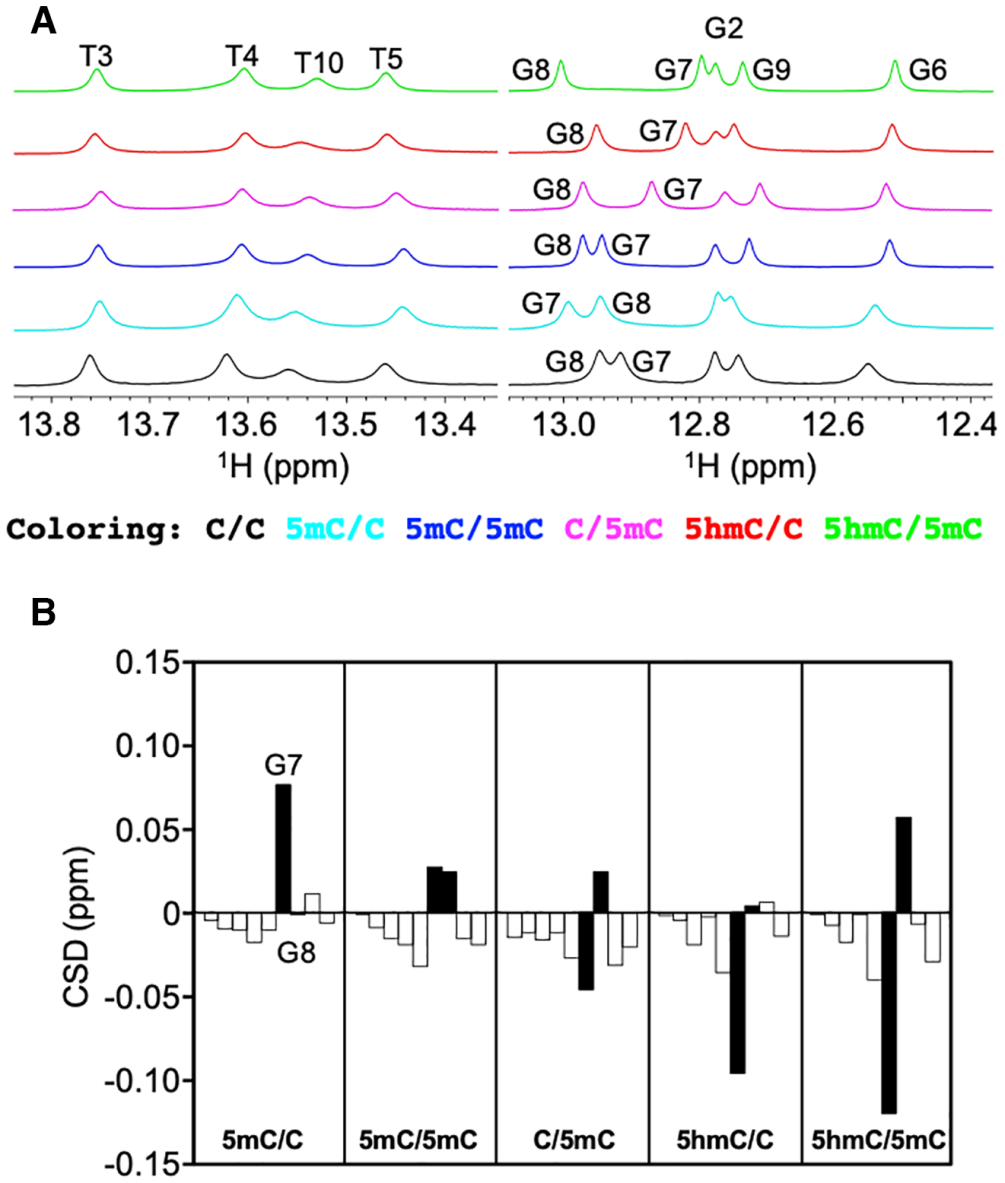
Dependence of imino proton chemical shifts on cytosine modification state. (**A**) Thymine and guanine imino proton regions in the 1D ^1^H NMR spectra of C/C (black), 5mC/C (cyan), 5mC/5mC (blue), C/5mC (magenta), 5hmC/C (red) and 5hmC/5mC (green). (**B**) Imino proton chemical shift differences (CSD) of the bases from G2 to T10. To obtain CSD, the chemical shifts of the modified dsDNA samples were subtracted from those of the unmodified dsDNA sample. The CSD values of G7 and G8 are shown as filled bars.

The ^1^H NMR spectra of the six dsDNA samples were measured, and the chemical shifts of the imino protons of the modified dsDNAs were compared with those of C/C to examine the effect of cytosine modification on dsDNA. Imino ^1^H resonances are usually well dispersed and isolated from other resonances of dsDNA, thereby facilitating analysis. Although cytosine does not contain an imino proton, we expected that the chemical shift of the guanine imino proton that forms a WC base pair with the (modified) cytosine would sense the cytosine modification state. Indeed, we observed differences in the imino proton chemical shifts among the six dsDNA samples, and the most pronounced chemical shift changes were confined to bases adjacent to the modified cytosine (Figures 3A, B). Interestingly, the imino ^1^H resonances of G7 in 5mC/C and 5mC/5mC, and G8 in 5mC/5mC, C/5mC and 5hmC/5mC were shifted downfield owing to cytosine methylation, whereas the imino ^1^H resonances of G7 in 5hmC/C and 5hmC/5mC were shifted upfield owing to cytosine hydroxymethylation. It seems likely that the former downfield shift is due to the electron-donating nature of the methyl group, while the latter upfield shift is due to the electron-withdrawing nature of the hydroxymethyl group. Both the electron-donating (increased shielding) and electron-withdrawing (decreased shielding) effects should be sensed by the guanine imino proton owing to its proximity to the hydrogen-bonded cytosine nucleotide. However, the magnitude of the chemical shift differences cannot be explained solely by the modification-induced changes in electron density in the vicinity of the imino proton. Collectively, therefore, these results infer that cytosine modification alters the chemical environment of the imino proton, reflecting changes either in the local conformation of the dsDNA or in the structural fluctuations around that conformation (structural dynamics).

### Cytosine modification affects the equilibrium of base-pair opening

Because cytosine modification is rather a small chemical change as compared with the size of the whole dsDNA molecule, we hypothesized that it might alter local structural flexibility. To test this hypothesis, we measured water–imino ^1^H exchange rates (*k*_1H_) by using the CLEANEX-PM experiment. If the stability of the hydrogen bond between the guanine imino proton and cytosine N3 is affected by the cytosine modification, the base-opening and closing rates (*k*_open_ and *k*_close_) of the GC base pair will change, resulting in alteration of *k*_1H_:

(4) $$\begin{equation*}{\rm{Closed\ }}\begin{array}{@{}*{1}{c}@{}} {\mathop \to \limits^{\ \ {k_{{\rm{open\ \ }}}}} }\\ {\mathop \leftarrow \limits_{\ \ {k_{{\rm{close}}}}\ \ } } \end{array}{\rm{\ Open\ }}\mathop \to \limits^{\ \ {k_{{\rm{int}}}}\ \ } {\rm{\ Exchange}},\end{equation*}$$

where *k*_int_ is the intrinsic ^1^H exchange rate of the open state.

**Figure 4.**
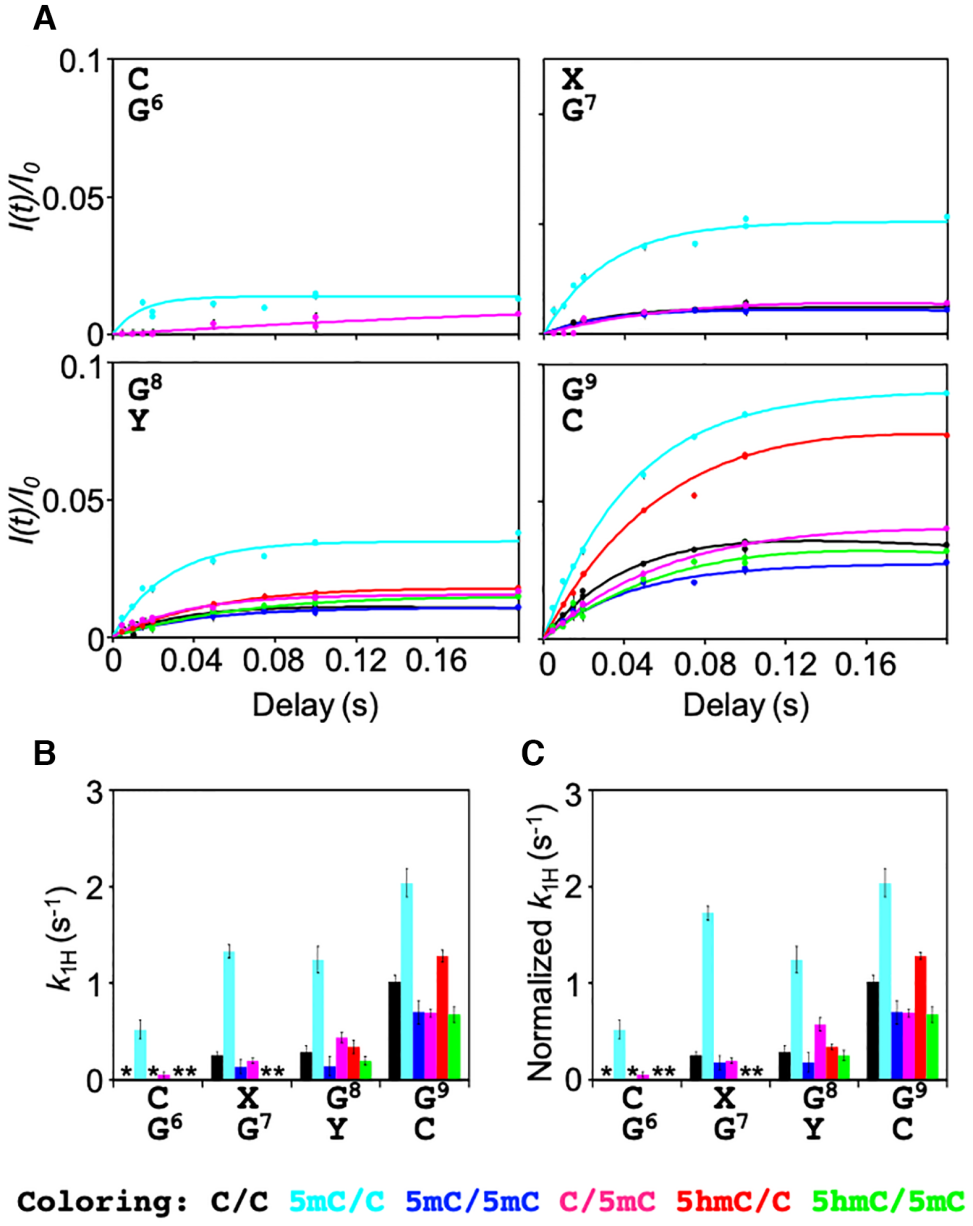
CLEANEX-PM experiments of imino protons in unmodified and cytosine-modified dsDNA. (**A**) CLEANEX-PM profiles for G6–G9 in C/C (black), 5mC/C (cyan), 5mC/5mC (blue), C/5mC (magenta), 5hmC/C (red), and 5hmC/5mC (green). Solid lines represent the best-fit lines to Equation 1. (**B**) ^1^H exchange rates *k*_1H_. Asterisks indicate the bases for which CLEANEX-PM signals were not detected. (**C**) Normalized *k*_1H_ rates. The *k*_1H_ rates of the guanine base-paired with 5mC were multiplied by 1.3.

As shown by representative CLEANEX-PM profiles in Figure 4A, water–imino ^1^H exchange was successfully detected. The intensity build-up was limited to values of *I*(*t*)/*I*_0_ < 0.1 even at the longest ^1^H exchange delay of 200 ms, indicating that the base-opening process is a very transient event (Figure 4A). Nevertheless, the *k*_1H_ rates of thymine were faster than those of guanine (as expected from the number of hydrogen bonds in AT and GC base pairs), indicating that changes in local hydrogen bonding capability can indeed be discerned by CLEANEX-PM (Supplementary Figure S3A). Although the highest CLEANEX-PM build-up values of *I*(*t*)/*I*_0_ for bases 6–9 around the modification sites were small, we observed differences in the *k*_1H_ rates depending on the state of cytosine epigenetic modification (Figure 4B). Interestingly, this epigenetic effect on the *k*_1H_ rates was not entirely local, but spread to both ends of the dsDNA, suggesting that a local base-opening process may be coupled to those of adjacent base pairs. Nevertheless, because both ends of dsDNA are intrinsically very flexible, a profound interpretation of the base-opening processes (i.e. the ^1^H exchange rates) at base pairs distal from the modified cytosine was beyond the scope of the present study. Instead, we focused on nucleotide positions 6–9, which include the modification sites themselves and the flanking base pairs, in order to explore the short-range effects caused by cytosine modification.

Because no catalyst was added to accelerate *k*_int_, the ^1^H exchange process can be assumed to be governed by the EX2 regime, in which *k*_close_ is much faster than *k*_int_ (16):

(5) $$\begin{equation*}{k_{1{\rm{H}}}} = {p_{{\rm{open}}}}{k_{{\rm{int}}}},\end{equation*}$$

where *p*_open_ (i.e. *k*_open_/[*k*_open_+*k*_close_]) denotes the population of the open state. *p*_open_ is usually further simplified as *K*_op_ (i.e. *k*_open_/*k*_close_) because *k*_open_ is much slower than *k*_close_ for ^1^H exchange of the guanine imino proton in dsDNA (16):

(6) $$\begin{equation*}{k_{1{\rm{H}}}} = {K_{{\rm{op}}}}{k_{{\rm{int}}}}\end{equation*}$$

^1^H exchange of the guanine imino proton is base-catalysed by the cytosine residue that forms the GC base pair, and *k*_int_ is proportional to 10^p*K*a(N3;C)–pKa(N1;G)^, in which pKa(N3;C) and pKa(N1;G) represent the pKa of N3 in cytosine and N1 in guanine, respectively. Recently, Dai and co-workers determined the p*K*_a_(N3;C) values of unmodified and epigenetically modified cytosine in dsDNA as 4.5 for cytosine, 4.4 for 5-methylcytosine, and 4.0 for 5-hydroxymethylcytosine (42). Using the p*K*_a_(N1;G) value of 9.4 (43), 10^p*K*a(N3;C)–pKa(N1;G)^ is calculated to be 1.3×10^–5^ for C, 1.0×10^–5^ for 5mC, and 0.40×10^–5^ for 5hmC. Because of this difference in *k*_int_ among the unmodified and modified cytosine nucleotides, we normalized the obtained *k*_1H_ rates of 5mC and 5hmC to that of C by multiplying by the factors 1.3 (i.e. 1.3×10^–5^/1.0×10^–5^) and 3.3 (i.e. 1.3×10^–5^/0.40×10^–5^), respectively. (Actually, ^1^H exchange of the guanine imino proton base-paired with 5hmC was not observed.) This normalization enabled us to interpret the resultant ^1^H exchange rate of each epigenetically modified GC base pair as the *K*_op_ value relative to that of the unmodified GC base pair (Equation 6; Figure 4C).

Strikingly, for nucleotide positions 6–9, the *k*_1H_ rates of 5mC/C were the fastest among the dsDNA samples examined (Figure 4C). For G6 in particular, ^1^H exchange was clearly detectable only for 5mC/C (and only slightly detectable for C/5mC). Moreover, the *k*_1H_ rates of 5mC/C were >4-fold higher for G7 and G8 compared with those of C/C. For the more distal G9, the effect was less pronounced; nevertheless, a 2-fold faster ^1^H exchange relative to unmodified dsDNA was still detected at this site. Similarly, hemi-methylation at position 8 (C/5mC) increased the *k*_1H_ rate of G8 compared with C/C. As outlined above (Equations 5 and 6), these changes in normalized *k*_1H_ value correspond to a relative increase in *K*_op_ or *p*_open_. Therefore, methylation of cytosine specifically on one strand of dsDNA seems to stabilize a more open conformation of the GC base pair. Interestingly, the increase in the *k*_1H_ rate of C/5mC was not as large as that of 5mC/C (Figure 4C), suggesting that the change in the *k*_1H_ rate due to epigenetic modification depends on its position along the DNA sequence.

Surprisingly, the *k*_1H_ rates at positions 6–9 of 5mC/5mC were comparable to those of C/C (Figure 4C). This indicated that, although methylation at position 7 (5mC/C) and position 8 (C/5mC) increased *K*_op_ at these particular base pairs (and in the vicinity in the case of hemi-methylation at position 7), this effect was reversed by further methylation at position 8 (5mC/5mC). Because hemi- and full methylation of cytosine at positions 7 and 8 had opposite effects on the *k*_1H_ rates, the chemical nature of the methyl group cannot be the sole determinant of the change in the base-opening equilibrium. Instead, it is most likely that cytosine methylation changes the conformational properties of dsDNA in a more complex manner, which manifests as differences in the measured *k*_1H_ rates.

In stark contrast, hydroxymethylation (5hmC/C) did not significantly alter *k*_1H_ at any of the positions examined, indicating that *K*_op_ was not changed by the presence of an additional hydroxymethyl group at the modified cytosine (position 7; Figure 4C). Further methylation at position 8 (5hmC/5mC) again decreased *k*_1H_, as observed for 5mC/5mC. Moreover, methylation at position 8 (5mC/5mC, C/5mC and 5hmC/5mC) decreased *k*_1H_ of position 9 compared with those of C/C. Taken together, the data show that, among the cytosine modifications examined, hemi-methylation at position 7 has the most marked effect on the base-opening equilibrium.

### Cytosine modification promotes the rate of local base-pair closing

The CLEANEX-PM experiments provided thermodynamic information on the base-opening and -closing process in the form of *K*_op_, but did not provide any kinetic information on the process, namely, the *k*_open_ or *k*_close_ rates. To characterize the effect of epigenetic cytosine modifications on the kinetics of dsDNA, we used a similar strategy but conducted on-resonance ^1^H *R*_1ρ_ relaxation dispersion experiments on the base-paired (guanine and thymine) imino proton. This type of experiment can determine conformational exchange rates on the microsecond-to-millisecond timescale (see Figure 2 for detailed information about the NMR pulse sequence used).

**Figure 5.**
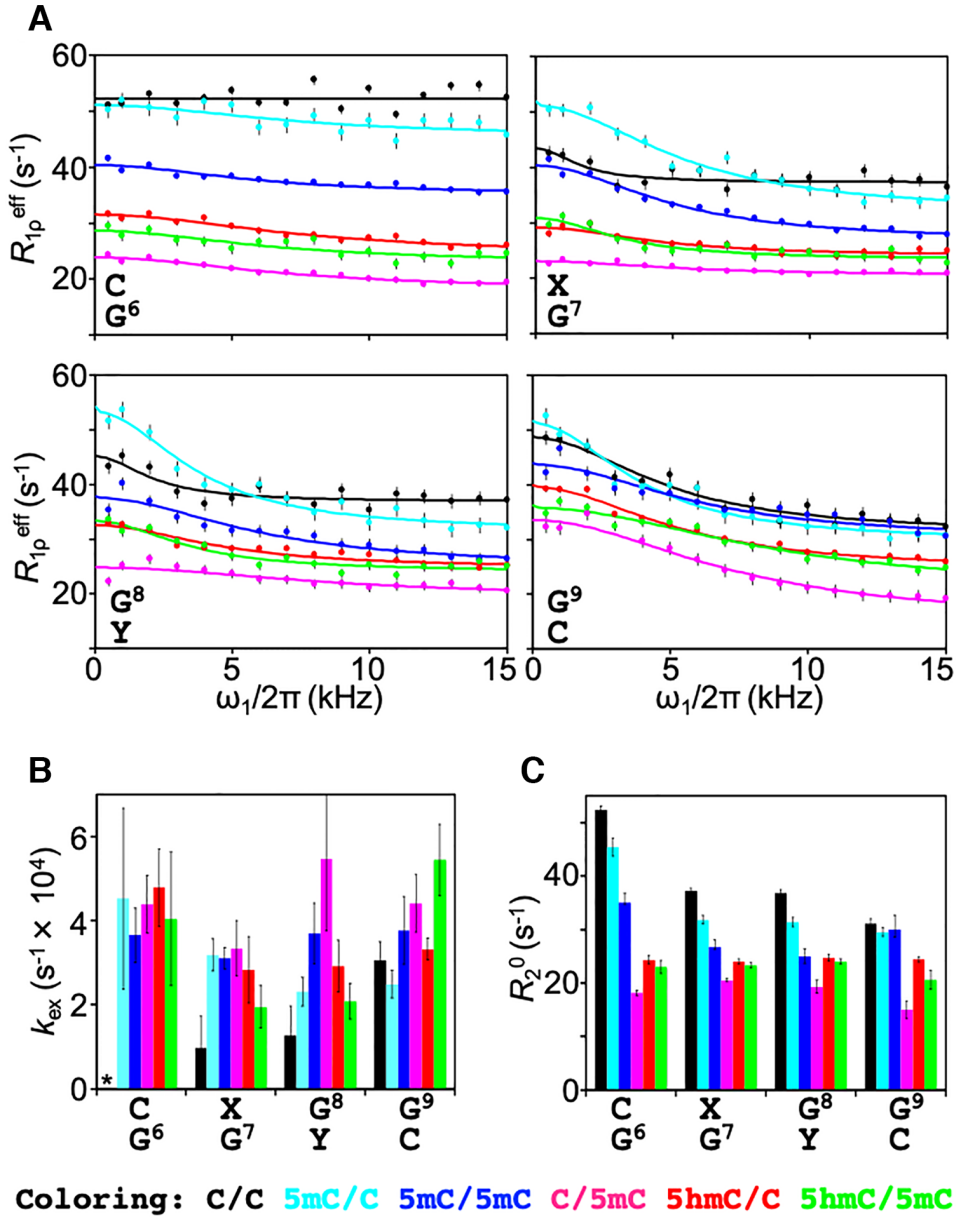
On-resonance *R*_1ρ_ dispersion experiments of imino protons in unmodified and cytosine-modified dsDNA. (**A**) On-resonance *R*_1ρ_ dispersion profiles for G6–G9 in C/C (black), 5mC/C (cyan), 5mC/5mC (blue), C/5mC (magenta), 5hmC/C (red) and 5hmC/5mC (green). Solid lines represent the best-fit lines to Equation 3. (**B**) The exchange rate, *k*_ex_, for G6–G9. No *R*_ex_ was observed for G6 in C/C, as indicated by the asterisk. (**C**) Intrinsic transverse relaxation rate, *R*_2_^0^, for G6–G9.

The resulting imino proton *R*_1ρ_ relaxation dispersion profiles indicated the presence of microsecond-to-millisecond conformational exchange in a manner dependent on the position of the respective base pairs in the DNA sequence (Figure 5A). Moreover, the contribution from conformational exchange (*R*_ex_) to the effective *R*_1ρ_ relaxation rates was larger for thymine than for guanine. This is in fine agreement with the expectation that the AT base pair will be less stable than the GC base pair owing to the smaller number of hydrogen bonds and thereby a comparably higher population of broken hydrogen bonds for AT, leading to a larger *R*_ex_ (Supplementary Figure S3B).

By fitting the *R*_1ρ_ relaxation dispersion data to Equation 3, we obtained the exchange rate, *k*_ex_ (i.e. *k*_open_+*k*_close_), of the base-opening process (Figure 5B) (18). Because the resultant *k*_ex_ rates were very fast, on the order of 10^4^ s^−1^, the conformational exchange of the dsDNA samples was categorized as fast exchange on the chemical shift timescale, and thus, *k*_ex_ could not be separated into *k*_open_ and *k*_close_ under these conditions. Owing to the high tendency of dsDNA to form WC base pairs (i.e., the high thermodynamic stability of the closed double-stranded form of DNA), however, *p*_open_ of a given base pair should be very small at our experimental temperature of 303 K. In other words, *k*_close_ (i.e. *p*_close_*k*_ex_) would be much faster than *k*_open_ (i.e. *p*_open_*k*_ex_). In such a situation, *k*_ex_ can be assumed to be almost equal to *k*_close_, meaning that a small change in *k*_open_ would not be reflected in *k*_ex_.

As in the CLEANEX-PM experiments, we focused again on the central base positions 6–9. For G6 in C/C, no *R*_1ρ_ relaxation dispersion (*R*_ex_ ≈ 0) was observed, and thus *k*_ex_ could not be derived. However, the samples containing cytosine modifications did show relaxation dispersion (Figure 5A): for example, the *k*_ex_ (≈*k*_close_) rates for G6 among the different samples were identical to each other within experimental error (Figure 5B), although ^1^H exchange for G6 had been observed only for 5mC/C and C/5mC (Figure 4). These results suggest that, although hemi-methylation at position 7 (5mC/C) and position 8 (C/5mC) increased *k*_open_ for G6 (observed by CLEANEX-PM), the tendency to return to the closed dsDNA structure was very similar among all dsDNA samples as reflected by the *k*_ex_ (≈ *k*_close_) values (observed by relaxation dispersion).

For nucleotide positions 7–9, all *k*_ex_ rates were remarkably similar to each other with some exceptions (Figure 5B). First, G7 and G8 of unmodified dsDNA (C/C) showed lower *k*_ex_ values as compared with all modified forms of dsDNA examined. Second, G8 of C/5mC and G9 of 5hmC/5mC showed higher *k*_ex_ rates than the corresponding positions of the other dsDNA samples. Together, these results indicate that both methylation and hydroxymethylation can increase *k*_close_; in other words, these modifications can shorten the lifetime of the open state (1/*k*_close_ ≈ 1/*k*_ex_). By CLEANEX-PM, however, we also observed significantly increased *k*_1H_ rates for G7 and G8 of 5mC/C and a slightly increased *k*_1H_ rate for G8 of C/5mC owing to hemi-methylation at positions 7 and 8, respectively. These results suggest that hemi-methylation at position 7 (5mC/C) increases both *k*_open_ and *k*_close_ for the base pairs flanking the methylation site (G6, G7, G8) whereas hemi-methylation at position 8 (C/5mC) increases both *k*_open_ and *k*_close_ only for the base pair itself (G8).

Interestingly, *R*_2_^0^ rates were also affected by the state of cytosine modification (Figure 5C). There are a few possible reasons to account for the variation observed in *R*_2_^0^ rates. First, the difference in the number of ^1^H nuclei in the dsDNA samples, which is related to the cross-relaxation rate, might explain variations in *R*_2_^0^. However, this seems unlikely because C/C, which has fewer ^1^H nuclei relative to the other samples, showed the fastest *R*_2_^0^ rates for all imino protons examined. Second, fast *R*_2_^0^ rates may originate from ^1^H exchange, but C/C, which showed the fastest *R*_2_^0^ rates, showed slower *k*_1H_ rates (Figure 4); thus, ^1^H exchange is not a cogent explanation. In addition to these two possibilities, we note that the intrinsic transverse relaxation rate *R*_2_^0^ is determined by both the overall rotational correlation time and the local flexibility on the picosecond-to-nanosecond timescale. In particular, the overall rotational correlation time is determined by the tertiary structure of a biomolecule. Therefore, the observed variations in *R*_2_^0^ infer that the tertiary structure and/or local flexibility differed among the six dsDNA samples.

### Cytosine modification alters local base-pair motion

Because neither tertiary structure nor local flexibility on the picosecond-to-nanosecond timescale can be captured by CLEANEX-PM or on-resonance imino ^1^H *R*_1ρ_ relaxation dispersion experiments, we carried out 1-μs MD simulations for each of the six dsDNA samples to examine their structure and flexibility (i.e. fluctuations around an average structure) and try to explain the variations in *R*_2_^0^.

First, we examined the radius of gyration of the dsDNA samples because this parameter affects the overall rotational correlation time of a molecule. However, the radius of gyration did not display a significant dependency on the state of cytosine modification (Supplementary Figure S4), suggesting that the differences in *R*_2_^0^ are not caused by variations in overall rotational correlation time. Next, we compared the local flexibility of the dsDNA samples by analysing the MD trajectories using the program 3DNA (34), which can extract intra-base-pair, inter-base-pair, and base-pair–axis parameters. These parameters were extracted from all the dsDNA atomic coordinates sampled every 10 ps over a 1-μs MD run. For each parameter set, we calculated the averages and standard deviations (SD) to compare the distribution of parameters among the different dsDNA samples.

**Figure 6.**
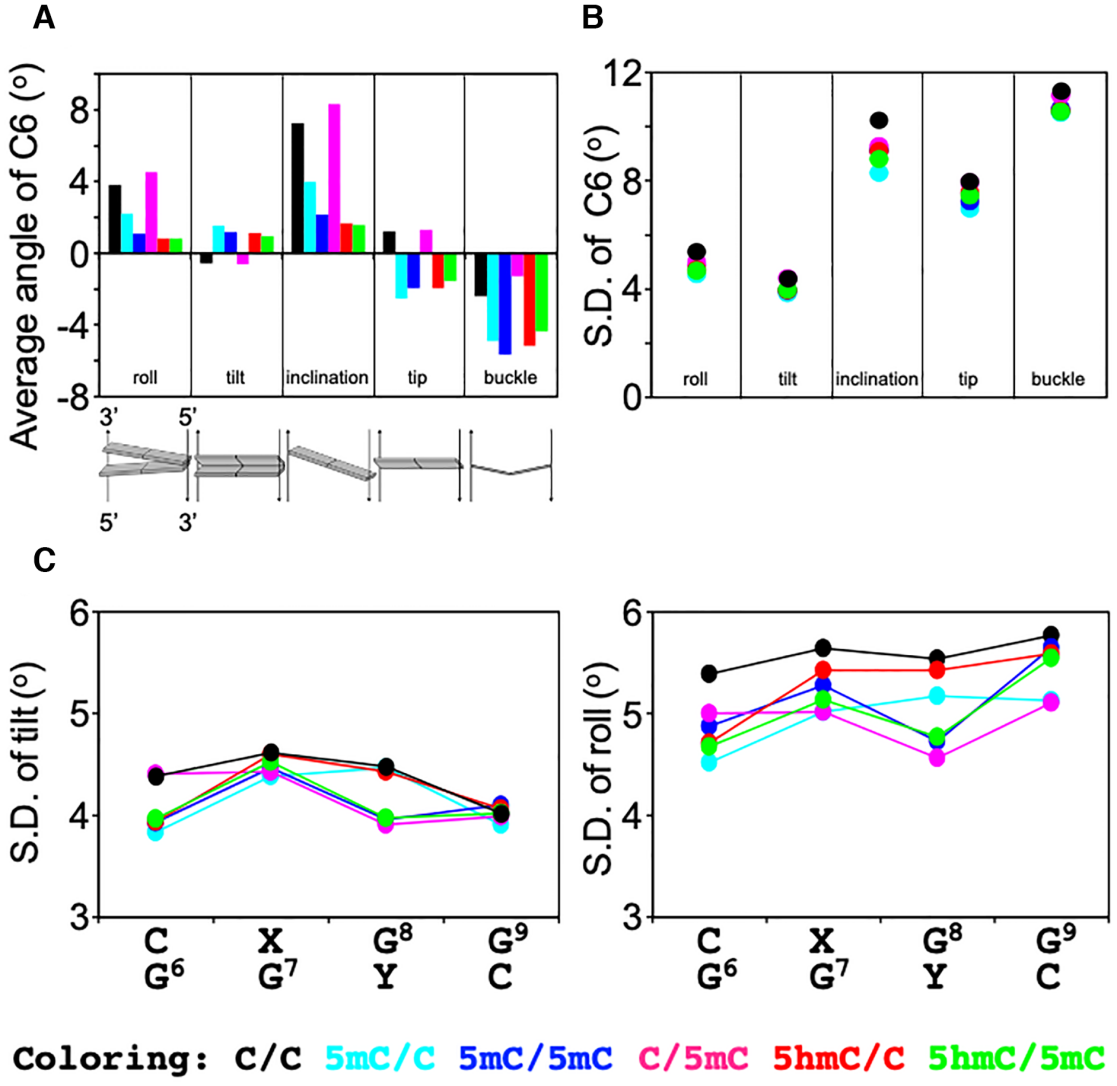
DNA helical parameters from MD simulations. (**A**) Average values of the roll, tilt, inclination, tip, and buckle angles for C6 in C/C (black), 5mC/C (cyan), 5mC/5mC (blue), C/5mC (magenta), 5hmC/C (red), and 5hmC/5mC (green) dsDNA. (**B**) Standard deviation of the tilt, roll, inclination, tip, and buckle angles for the C6:G base pair. (**C**) Standard deviation of the tilt and roll angles for C6-G9.

For most parameters, the derived average values were almost identical at all base pairs among all dsDNA samples, with the exception of the C6:G base pair (Figure 6 and Supplementary Figure S5). At this position, the average angles of roll, tilt, inclination, tip, and buckle were found to differ between unmodified and modified dsDNA except for C/5mC (Figure 6A), suggesting that these angles (at position 6) depend on the cytosine modification state of position 7 (among these samples only C/C and C/5mC are unmodified at position 7). The roll and tilt angles are defined in terms of local stacking configurations at successive base pairs, and are sensitive to, for example, the degree of DNA curvature. The inclination and tip angles reflect the orientation of the base-pair planes with respect to the helical axis, and the buckle angle measures deviations in planarity in a base pair. The average roll angles of C/C and C/5mC were larger than those of the other modified dsDNA samples, indicating that the C6:G base pairs in C/C and C/5mC are curved toward the major groove as compared with the other modified dsDNA samples. In addition, C/C and C/5mC showed negative values in the average tilt angle, whereas the other modified dsDNA samples displayed positive values, indicating that C/C and C/5mC are on average slightly curved at C6 in the opposite direction relative to the other modified dsDNA samples. Similar differences were observed for the average inclination and tip parameters, likewise depending on the modification state at position 7. Although the differences in the average angular parameters were only a few angular degrees, such changes in various angular parameters might have a significant effect on the local structure when taken together. Additional factors essentially untraceable by molecular dynamics, such as differences in tautomeric reactivity (44) occurring either classically over an activation energy-barrier or quantum-mechanically by tunnelling of hydrogen atoms along hydrogen bonds (45–47) that accompany changes in base-pair geometry may further contribute to the epigenetically distinct dynamics. Over longer timescales, these small differences may build up into larger distinctions between the different dsDNA samples that ultimately give rise to different dynamics, as experimentally observed by solution NMR.

In terms of the SD of the derived angular parameter distributions, which approximately represents fluctuations around the average local structure of the dsDNA over the course of the MD trajectories, the SD in the roll, tilt, inclination, tip, and buckle angles differed especially between the unmodified and modified dsDNA samples. Similar to the average values, the differences were pronounced for the C6:G base pair, where the unmodified dsDNA displayed larger SD values (i.e. broader distributions) as compared with the modified dsDNA (Figure 6B). In a comparison of the SD values of each parameter along the sequence, the SD values of tilt and roll angles from the C6:G to G9:C base pairs were smaller in the modified dsDNA than in the unmodified dsDNA (Figure 6C), indicating less flexibility at these base pairs in the modified dsDNA sample. In addition, all dsDNA samples containing methylation at position 8 (5mC/5mC, C/5mC and 5hmC/5mC) showed smaller SD values of tilt and roll angles of G8:C than the dsDNA samples unmethylated at position 8 (C/C, 5mC/C and 5hmC/C). Taken together, therefore, these results suggest that cytosine methylation and hydroxymethylation can decrease local flexibility in the dsDNA structure.

## DISCUSSION

We have shown that site-specific cytosine modification alters local dynamics rather than the overall thermal stability of DNA. We have also demonstrated that the dsDNA dynamics altered by cytosine modification can be semi-quantitatively analysed by combining data from CLEANEX-PM and imino ^1^H *R*_1ρ_ relaxation dispersion experiments in a complementary way. Using this approach, we characterized the base-opening (*k*_open_) and base-closing (*k*_close_) processes and found that only hemi-methylation (5mC/C) at a single nucleotide site (position 7 of our sample) significantly increased both the *k*_open_ and *k*_close_ rates. By contrast, the other cytosine modifications examined (5mC/5mC, C/5mC, 5hmC/C and 5mC/5hmC) increased *k*_close_ but did not change *k*_open_ significantly. Because all the p*K*_a_(N3;C) values of modified cytosine N3 are lower than that of unmodified cytosine N3 (4.5 for cytosine, 4.4 for 5-methylcytosine, and 4.0 for 5-hydroxymethylcytosine), the N3 atoms of modified cytosine should be less protonated at neutral pH as compared with unmodified cytosine. In turn, this should be unfavourable for formation of the HG base pair because protonation of cytosine N3 is required to stabilize an HG GC base pair. Therefore, it seems likely that the increase in *k*_close_ represents either destabilization of an HG base pair or a shorter lifetime of an HG base pair (proportional to 1/*k*_close_).

Previously, it was reported that the *k*_ex_ of WC to HG conformational exchange is on the order of 10^3^ s^−1^ by ^13^C and ^15^N *R*_1ρ_ relaxation dispersion (13,19–22). Most of those studies were conducted under low pH conditions because lower pH enhances the protonation of cytosine N3, which stabilizes the HG GC base pair. The *k*_ex_ rates obtained in the present study were on the order of 10^4^ s^−1^, which is faster by one order of magnitude than the previously determined *k*_ex_ rates. This difference arises because the dsDNA dynamics were analysed at neutral pH, at which the closing kinetics (*k*_close_) should be much faster relative to lower pH. Moreover, the ^1^H *R*_1ρ_ relaxation dispersion experiment used in the present study can detect faster motions as compared with the previously used ^13^C and ^15^N *R*_1ρ_ relaxation dispersion experiments. An alternative possibility is that the HG GC base pair, which has two inter-base hydrogen bonds, is not perfectly formed at neutral pH, and our ^1^H *R*_1ρ_ relaxation dispersion measurements detected conformational exchange between the WC base pair and such a state.

In the present study, the increase in the *k*_open_ rate of 5mC/C (Figure 4C) was particularly striking, and indicates that the WC base pairs of C6:G to G9:C are destabilized by cytosine methylation. Destabilization of the WC base pair cannot be explained simply by the lower pKa(N3;C) value. The MD simulations indicated that the dynamics of local curvature and the flexibility of modified dsDNA differs from that of unmodified dsDNA. Therefore, local changes in structure and dynamics of dsDNA induced by cytosine modification may cause destabilization of the WC base pair. In addition, the destabilization of the WC base pair appears to also depend on the DNA sequence as we obtained the different *k*_open_ rates between 5mC/C and C/5mC. This is consistent with a previous study by Alvey *et al.* who also reported that dsDNA stability depends on the position of modification (21).

UHRF1 binds selectively to hemi-methylated dsDNA (5mC/C), but not to unmethylated (C/C) or fully methylated (5mC/5mC) dsDNA (7). As shown in the present study, the global stability of the dsDNA samples was identical irrespective of the cytosine modification state of dsDNA; however, local dynamics was altered by cytosine modification and especially by hemi-methylation of dsDNA (Figure 5). In particular, the *k*_1H_ rates of 5mC/C were markedly different from those of all the other dsDNA samples, including unmodified and fully methylated dsDNA (Figure 4). Thus, UHRF1 may exploit such differences in local dynamics to selectively bind to and thereby recognize hemi-methylated DNA.

During preparation of this manuscript, it was reported that UHRF1 binds to fully carboxyl-methylated dsDNA (5caC/5caC) with high affinity (48). Therefore, we additionally prepared this type of dsDNA and analysed its structural dynamics by the CLEANEX-PM and imino ^1^H *R*_1ρ_ relaxation dispersion experiments. As a result, the imino ^1^H resonances of G7 and G8 in 5caC/5caC were shifted upfield, which is reasonable because the carboxyl group has the electron-withdrawing nature (Supplementary Figures S6B, C). Unexpectedly, ^1^H exchange and *R*_1ρ_ relaxation dispersion of G7 and G8 were not observed (Supplementary Figure S6D), indicating that 5caC/5caC is more stable than C/C. In fine accordance with these NMR data, we observed that the melting temperature of 5caC/5caC was higher than that of C/C (58.84 ± 0.02°C versus 55.37 ± 0.09°C). Therefore, UHRF1 may recognize 5caC/5caC in a different manner from 5mC/C. Because structural data of a UHRF1–5caC/5caC complex are not yet available, it however remains elusive what the exact differences are that UHRF1 exploits to discriminate fully carboxyl-methylated from hemi-methylated DNA.

5-Methylcytosine and 5-hydroxymethylcytosine, which are found in most plants, animals, and fungi, have profound effects on genomic stability, gene expression, and development (1,2). Local changes in conformation and dynamics of dsDNA due to cytosine modification are therefore likely to play an important role in all these phenomena. In a physiological (nuclear) context, intriguingly it has been reported that HG base pairs are stabilized under conditions of molecular crowding, whereas WC base pairs are destabilized (49). This infers that, relative to in vitro experiments, dsDNA dynamics may be amplified in living cells, which are highly crowded environments. It seems that DNA-binding proteins including UHRF1 might exploit such dsDNA dynamics in their search for specific target DNA sequences.

For mice it has been shown that in addition to hemi-methylated dsDNA, hemi-hydroxymethylated/hemi-methylated dsDNA also exists at high levels in various tissues including kidney, heart, liver, and brain (cerebellum) (50). In this context, it is highly intriguing that we observed that 5mC/5mC and 5hmC/5mC exhibited different structural dynamics, in particular: *k*_ex_ of G7 and G8 (Figure 5B). Such differences in local dynamics might be exploited by proteins (by modulating the association or dissociation rates of binding to these specifically modified types of DNA), such as methyl-CpG-binding domain proteins or TET, to discern one epigenetic state from the other. Therefore, in addition to the specific case of UHRF1, it will be interesting to study structural dynamics of epigenetically (un)modified dsDNA in the presence of these epigenetic mark reading proteins in the future.

## Supplementary Material

gkaa1210_Supplemental_File

## Notes

Present address: Kenji Sugase, Department of Molecular Engineering, Graduate School of Engineering, Kyoto University, Kyoto-Daigaku Katsura, Nishikyo-Ku, Kyoto 615-8510, Japan.
