# Supplementary material for: Structural dynamics of double-stranded DNA with epigenome modification
Source: Nucleic Acids Res. 2020 Dec 18;49(2):1152–62. doi: 10.1093/nar/gkaa1210 (PMC7826269; doi:10.1093/nar/gkaa1210)
Supplement: gkaa1210_Supplemental_File [file gkaa1210_supplemental_file.pdf]

Supplementary Data

Structural dynamics of double-stranded DNA modulated by epigenome modification

Ayako Furukawa[1,2], Erik Walinda[3], Kyohei Arita[1], and Kenji Sugase[2,4*]

[1] Graduate School of Medical Life Science, Yokohama City University, 1-7-29 Suehiro-cho, Tsurumi-ku, Yokohama, Kanagawa 230-0045, Japan

[2] Bioorganic Research Institute, Suntory Foundation for Life Sciences, 8-1-1 Seikadai, Seika, Soraku, Kyoto 619-0284, Japan

[3] Department of Molecular and Cellular Physiology, Graduate School of Medicine, Kyoto University, Yoshida Konoe-cho, Sakyo-ku, Kyoto 606-8501, Japan

[4] Department of Molecular Engineering, Graduate School of Engineering, Kyoto University, Kyoto-Daigaku Katsura, Nishikyo-Ku, Kyoto 615-8510, Japan

This file contains:

Supplementary method

Supplementary Figures S1-S6

Supplementary Table S1-S2

Supplementary method

The influence of cross-relaxation on ^{1}H $R_{1\rho}$ relaxation dispersion

For ^{15}N relaxation experiments such as R_1, R_2, and $R_{1\rho}$ rate measurements, the contribution of ^{15}N-^{15}N cross-relaxation to effective relaxation rates is usually negligible because of its small gyromagnetic ratio and the relatively long distances between adjacent ^{15}N atoms in proteins and nucleic acids. For the ^{1}H $R_{1\rho}$ relaxation experiment conducted in this study, however, ^{1}H-^{1}H cross-relaxation in the rotating frame, known as the rotating-frame Overhauser effect (ROE), must be taken into account because it may change the effective ^{1}H $R_{1\rho}$ relaxation rate (${}^{\mathrm{eff}}R_{1\rho} = R_2^0 + R_{\mathrm{ex}}$). Because proton density is lower in DNA than in protein, the contribution of ^{1}H-^{1}H cross-relaxation in the rotating frame to ^{1}H $R_{1\rho}$ relaxation should be smaller in DNA. To our knowledge, however, no theoretical examination of this matter has been conducted. Below, we have simulated the ^{1}H $R_{1\rho}$ relaxation of imino protons of guanine and thymine in the double-strand DNA (dsDNA) sample, including the effect of ^{1}H-^{1}H cross-relaxation.

In the case of an *n*-spin system, a set of coupled differential equations describes the time evolution of the magnetization of the individual spins. The matrix form of the differential equations can be written as (1):

$$\frac{\mathrm{d}\mathbf{M}(t)}{\mathrm{d}t} = -\mathbf{R}\mathbf{M}(t)$$

, where $\mathbf{M}(t)$ is the column vector with^{1}H peak intensities after a spin-lock time of t. $\mathbf{R}$ is the matrix that contains auto-relaxation rates, ρ_{ii}, as the diagonal elements; and cross-relaxation rates, $\sigma^{\mathrm{ROE}}{}_{ij}$, as the non-diagonal elements. Because matrix $\mathbf{R}$ does not contain the terms that describe chemical exchange, the amplitude of the relaxation due to chemical exchange, R_{ex}, cannot be simulated. However, cross-relaxation does not alter R_{ex}, which is determined by the exchange rate, populations, chemical shift differences between the interconvertible states, and the offset from the carrier frequency (2). These parameters are independent of cross-relaxation. In the simulation, therefore, we focused only on the contribution of cross-relaxation to the intrinsic relaxation rate, R_2^0.

For each pair of ^{1}H-^{1}H 2-spin systems, σ^{ROE} can be calculated by using the following equations under the assumption that dsDNA is treated as a cylinder shape molecule and undergoes axially symmetric rotation around the cylinder axis (1,3):

$$\sigma^{\mathrm{ROE}} = \frac{h^2 \mu_0^2 \gamma_{\mathrm{H}}^4}{256\pi^4 r^6}[2J(0) + 3J(\omega_{\mathrm{H}})]$$

$$J(0) = \frac{2}{5} S^2 \sum \mathrm{A}_k \tau_k$$

$$J(\omega_H) = \frac{2}{5}\frac{S^2 \sum A_k \tau_k}{1 + \omega_H^2 + \tau_k^2}$$

, where h is the Planck constant, μ_0 is the vacuum permeability, γ_H is the gyromagnetic ratio for ^{1}H, and r is the interatomic distance between an imino proton of interest and another proton (see below). S^2 denotes the generalized order parameter, which represents the rigidity of a H-N (or H-C) bond vector; S^2 ranges from 0 to 1, but here we set S^2 as 0.8, which is a typical S^2 value for dsDNA (4,5). A_k and τ_k (k = 1, 2, and 3) are defined as $A_1 = (3\cos^2\theta - 1)^2/4$, $A_2 = 3\sin^2\theta\cos^2\theta$, $A_3 = 3\sin^4\theta/4$, $\tau_1 = (6D^{\perp})^{-1}$, $\tau_2 = (5D^{\perp} + D^{\parallel})^{-1}$, and $\tau_3 = (2D^{\perp} + 4D^{\parallel})^{-1}$. Here, θ is the angle between the H-N (or H-C) bond vector and the cylinder axis. Because the DNA base planes are approximately perpendicular to the cylinder axis, θ is set to 90˚. The base planes are actually 1.2˚ inclined; however, this small deviation does not change σ^{ROE}, as far as we tested. $D^{\perp}$ and $D^{\parallel}$ are the perpendicular and parallel rotational diffusion constants, respectively. dsDNA can be treated as a cylinder shape molecule with length L (number of base pairs × 3.38 Å), diameter d (~20 Å), and aspect ratio p ($p = L/d$). For this type of cylinder, $D^{\parallel}$ and $D^{\perp}$ are calculated by using the equations (6):

$$D^{\perp} = \frac{3kT(\ln p - 0.662 + 0.917/p - 0.050/p^2)}{\pi\eta_0 L^3}$$

$$D^{\parallel} = \frac{4kTp^2}{3.84\pi\eta_0 L^3(1 + 0.677/p - 0.183/p^2)}$$

, where k is the Boltzmann constant, T is the absolute temperature, and η_0 is the viscosity of the solvent.

Regarding the interatomic distance, r, we measured distances from imino protons of interest to other protons in the structure of the dsDNA sample used in the present study. For imino protons in guanine, the most adjacent nonlabile proton was the imino proton in the preceding or succeeding base. For those in thymine, H2 in adenine that is base-paired with the thymine under analysis was the nearest. The distances used in the σ^{ROE} calculations are summarized in Table S1. It should be noted that resonances of adenine H2 are usually separate from those of imino protons: according to the Biological Magnetic Resonance Data Bank (http://www.bmrb.wisc.edu/), the averaged chemical shifts of adenine H2, the imino proton in guanine (guanine H1), and the imino proton in thymine (thymine H3) are 7.65, 11.88, and 13.00 ppm, respectively. In the ^{1}H $R_{1\rho}$ relaxation experiments conducted in this study, the carrier frequency was set to one of the imino proton resonances. Therefore, the effective magnetic field at the resonance frequency of adenine H2 is tilted at a weak spin-lock power, ω_1, and thus σ^{ROE} is scaled by $\sin\alpha$ (Supplementary Figure S1). Here, $\alpha = \tan^{-1}(\omega_1/\Omega)$ and Ω is the chemical shift difference between the spin-lock carrier frequency and the resonance frequency of adenine H2 (7). Although the contribution of σ^{ROE} to the effective ^{1}H $R_{1\rho}$ relaxation rate depends on ω_1, here we calculated σ^{ROE} with $\sin\alpha = 1$ to understand its maximum contribution to the effective ^{1}H $R_{1\rho}$ relaxation rate.

The parameter values used in the simulation of ^{1}H $R_{1\rho}$ relaxation were as follows: $h = 6.626 \times 10^{-34}$ J·s, $\mu_0 = 1.257 \times 10^{-6}$ N·A^{-2}, $\gamma_H = 2.675 \times 10^{8}$ T^{-1}·s^{-1}, $\omega_H = 3.770 \times 10^{9}$ rad·s^{-1}, $S^2 = 0.8$, $\theta = 90°$, $L = 40.56$ Å, $d = 20.00$ Å, $p = 2.03$, $k = 1.381 \times 10^{-23}$ J·K^{-1}, $\eta_0 = 7.980 \times 10^{-4}$ P·s, $T = 303.15$ K, r (Supplementary Table S1), and sinα = 1. The calculated σ^{ROE} values are also listed in Table S1. The auto-relaxation rate ρ was set to R_2^0 values determined by the ^{1}H $R_{1\rho}$ relaxation experiments. For ^{1}H resonances for which R_2^0 values were not available, we set R_2^0 to 10, 15, 20, 25, 30, 35, 40, and 45 s^{-1}, and examined how much the contribution of σ^{ROE} to ^{1}H $R_{1\rho}$ relaxation rate changed.

We constructed a 17 × 17 matrix **R** composed of ρ and σ^{ROE}, and simulated the time evolution of the 17 ^{1}H resonances listed in Supplementary Table S1 by using the program pari-gp (https://pari.math.u-bordeaux.fr/). As a result, magnetization decay profiles calculated with σ^{ROE} were found to be almost identical to those without σ^{ROE} (Supplementary Figure S2). The deviations between the decay profiles with and without σ^{ROE} were very small, but those for thymine were slightly larger than those for guanine because of the cross-relaxation from adenine H2, which is closest to the imino proton in thymine (Supplementary Table S1). The resultant effective ^{1}H $R_{1\rho}$ relaxation rates calculated with σ^{ROE} were in a good agreement with those without σ^{ROE} (Supplementary Table S2) within the experimental error (Figure 5). The effective ^{1}H $R_{1\rho}$ relaxation rates are not significantly dependent on the R_2^0 values, but the differences between the rates with and without σ^{ROE} become smaller as R_2^0 increases.

In conclusion, we have shown by the simulation that the contribution of ^{1}H-^{1}H cross-relaxation to the effective ^{1}H $R_{1\rho}$ relaxation rates is negligible in the case of the dsDNA sample used in the present study, especially for the imino proton in guanine. However, the contribution of ^{1}H-^{1}H cross-relaxation depends on auto-relaxation rates, which are closely correlated with molecular weight or the number of base pairs. Therefore, it is recommended that a similar simulation should be performed for each dsDNA sample.

References

1. Peter,C., Daura, X. and van Gunsteren,W.F. (2001) Calculation of NMR-relaxation parameters for flexible molecules from molecular dynamics simulations. J. Biomol. NMR, 20, 297-310.
2. Korzhnev,D.M., Orekhov,V.Y. and Kay,L.E. (2005) Off-resonance $R_{1\rho}$ NMR studies of exchange dynamics in proteins with low spin-lock fields: an application to a Fyn SH3 domain. J. Am. Chem. Soc., 127, 713-721.
3. Tjandra,N., Feller,S.E., Pastor,R.W. and Bax,A. (1995) Rotational diffusion anisotropy of human ubiquitin from ^{15}N NMR relaxation. J. Am. Chem. Soc., 117, 12562-12566.
4. Spielmann, H.P. (1998) Dynamics in psoralen-damaged DNA by ^{1}H-detected natural abundance ^{13}C NMR spectroscopy. Biochemistry, 37, 5426-5438.

5. Taranova,M., Hirsh,A.D., Perkins,N.C. and Andricioaei,I. (2014) Role of microscopic flexibility in tightly curved DNA. J. Phys. Chem. B, 118, 11028-11036.
6. Ortega,A. and García de la Torre,J. (2003) Hydrodynamic properties of rodlike and disklike particles in dilute solution. J. Chem. Phys., 119, 9914-9919.
7. Cavanagh,J., Fairbrother,W.J., III, Palmer,A.G.3rd, Rance,M. and Skelton,N.J. (2006) Protein NMR Spectroscopy, Second Edition: Principles and Practice. Academic Press.

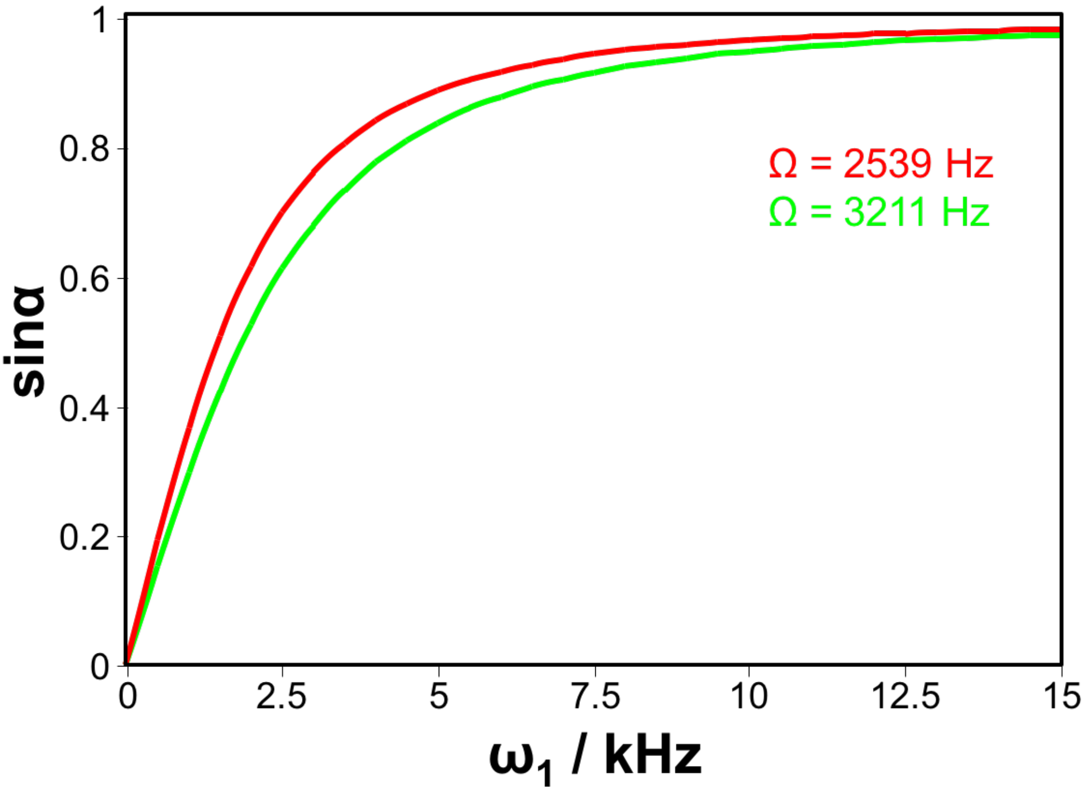

Supplementary Figure S1. Dependence of spin-lock power ω_1 on sinα for the chemical shift difference between adenine H2 and thymine H3 (red), and between adenine H2 and guanine H1 (green). The chemical shifts of adenine H2, guanine H1, and thymine H3 were 7.65, 11.88, and 13.00 ppm, respectively. Ω was calculated for the ^{1}H magnetic field of 600 MHz.

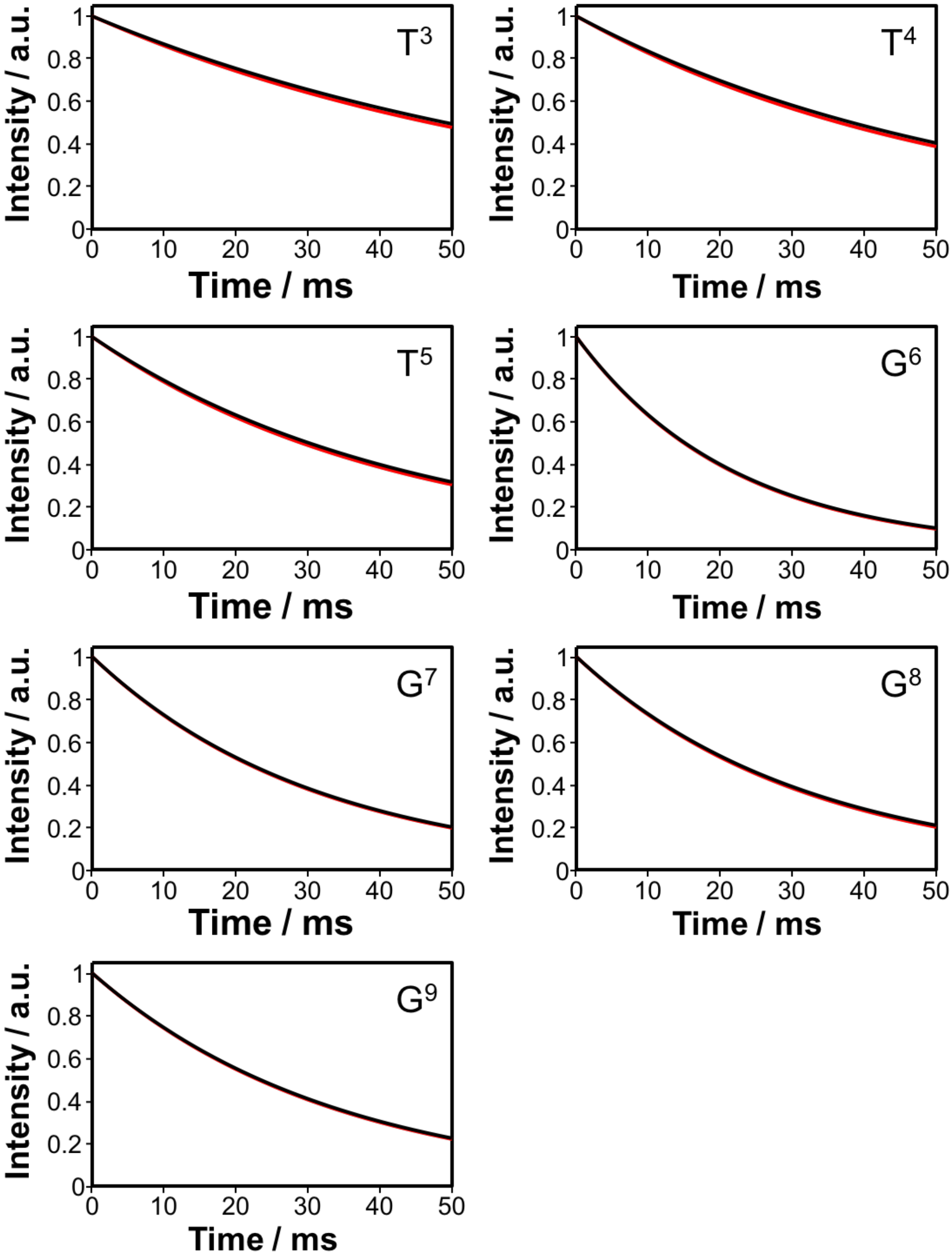

Supplementary Figure S2. Representative simulation results of imino proton decays in dsDNA with 12 base pairs. Simulations were conducted with σ^{ROE} (red) and without σ^{ROE} (black).

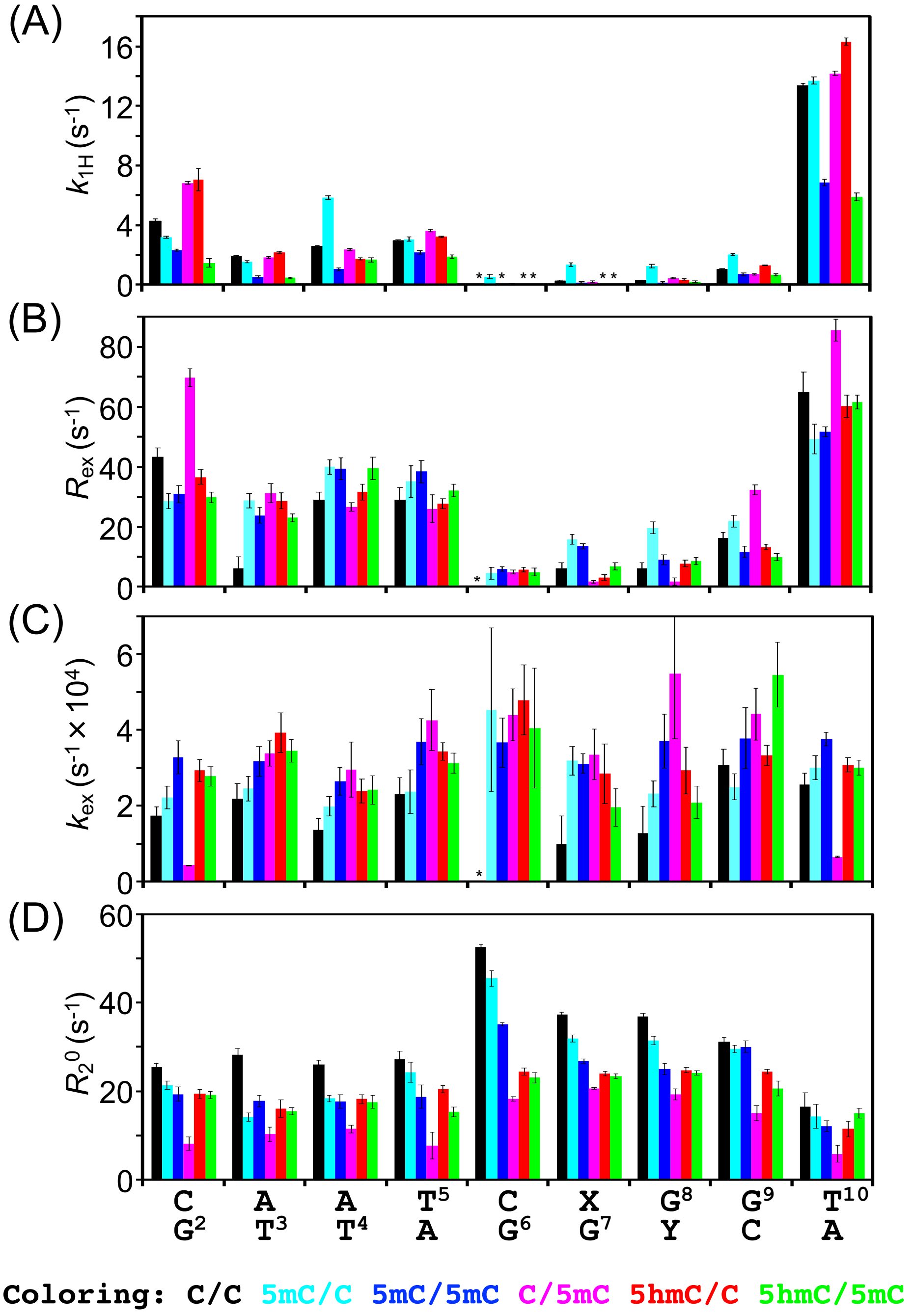

Supplementary Figure S3. (A) The ^{1}H exchange rates k_{1H} for G2–T10 in C/C (black), 5mC/C (cyan), 5mC/5mC (blue), C/5mC (magenta), 5hmC/C (red), and 5hmC/5mC (green). Asterisks indicate the bases for which CLEANEX-PM signals were not detected. (B) Relaxation rates, R_{ex}, for G2–T10. No R_{ex} was observed for G6 in C/C, as indicated by the asterisk. (C) Exchange rates, k_{ex}, for G2–T10. No R_{ex} was observed for G6 in C/C, as indicated by the asterisk. (D) Intrinsic transverse relaxation rate, R_2^0, for G2–T10. The colour scheme of (B), (C) and (D) is the same as (A).

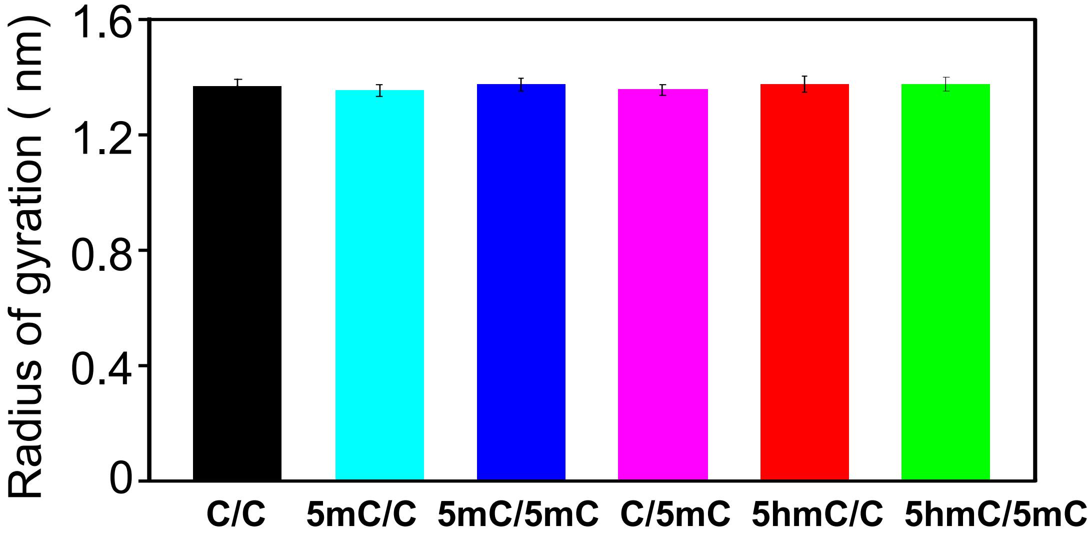

Supplementary Figure S4. Average radius of gyration of dsDNA during the 1-μs MD simulations for C/C (black), 5mC/C (cyan), 5mC/5mC (blue), C/5mC (magenta), 5hmC/C (red), and 5hmC/5mC (green). Error bars indicate the standard deviation in the radius of gyration taken over all time frames of the trajectory.

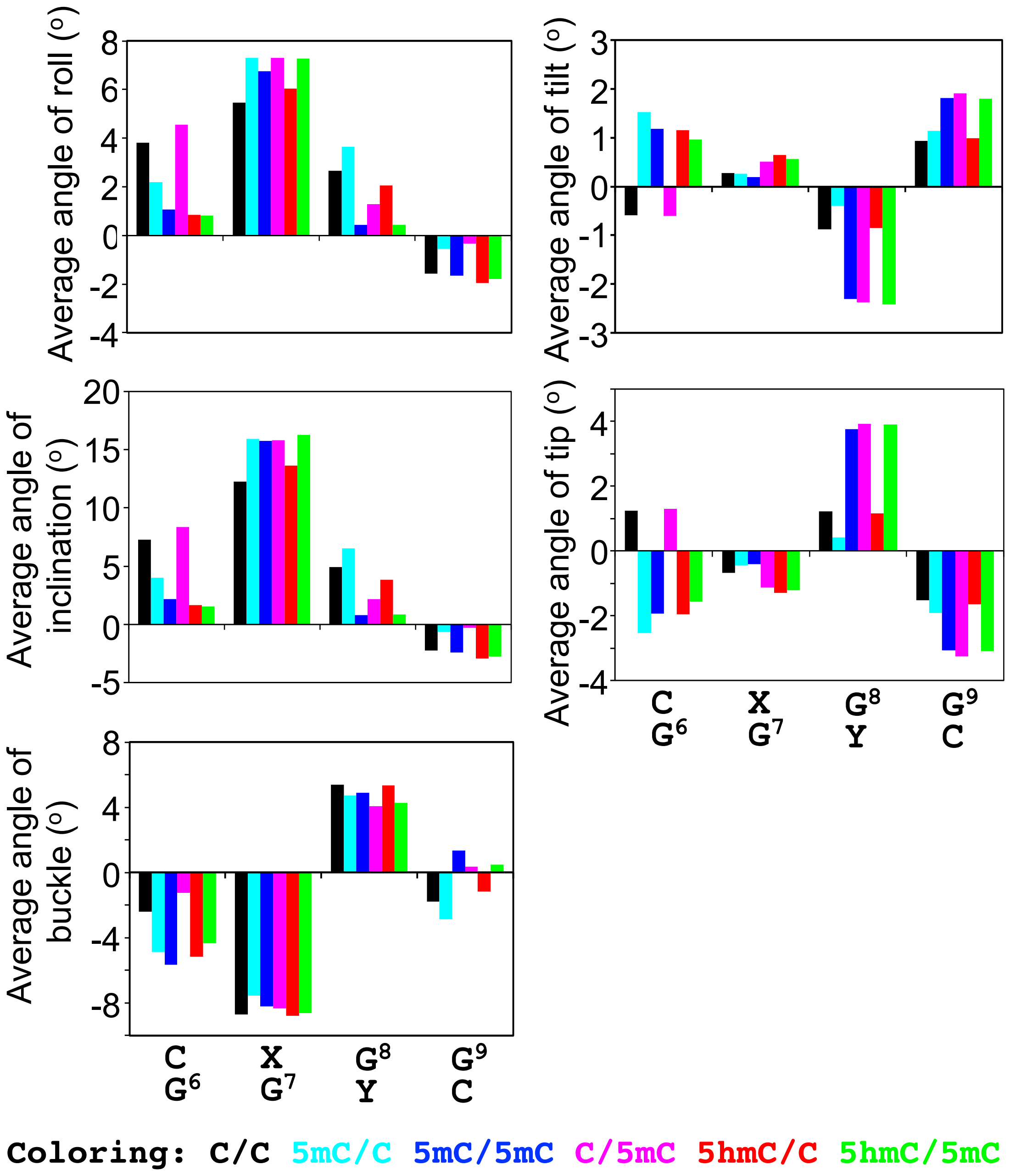

Supplementary Figure S5. Average angle at C6:G to G9:C base pairs for C/C (black), 5mC/C (cyan), 5mC/5mC (blue), C/5mC (magenta), 5hmC/C (red), and 5hmC/5mC (green).

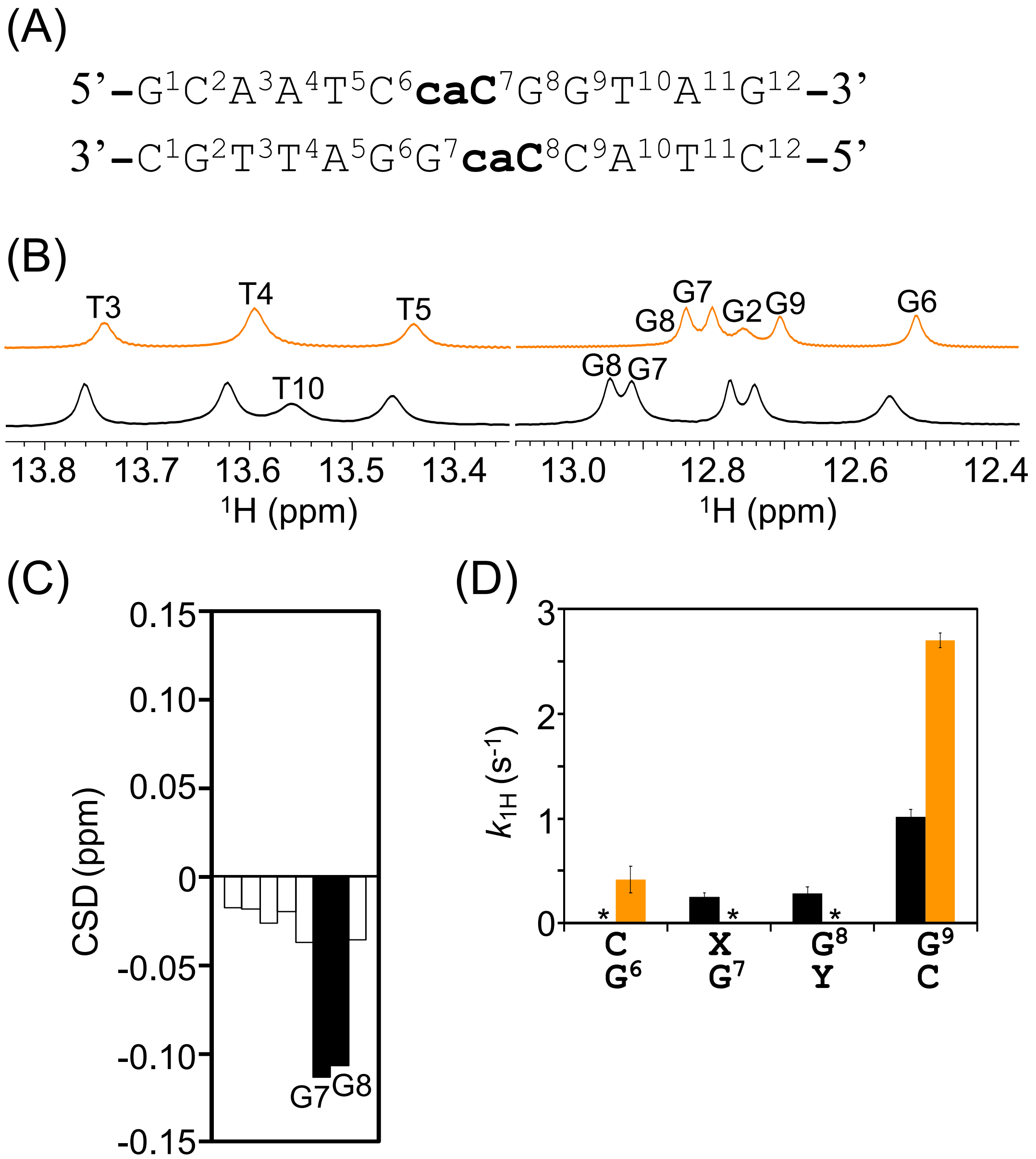

Coloring: C/C 5caC/5caC

Supplementary Figure S6. Fully carboxyl-methylated dsDNA (A) The dsDNA sequence. (B) Thymine and guanine imino proton regions in the 1D ^{1}H NMR spectra of C/C (black) and 5caC/5caC (orange). (C) Imino proton chemical shift differences (CSD) of the bases from G2 to T10. To obtain CSD, the chemical shifts of the 5caC/5caC dsDNA samples were subtracted from those of the unmodified dsDNA sample. The CSD values of G7 and G8 are shown as filled bars. (D) ^{1}H exchange rates k_{1H}. Asterisks indicate the bases for which CLEANEX-PM signals were not detected.

Supplementary Table S1. Distance from imino protons to other protons and calculated σ^{ROE}

		Distance (Å)[a]																
		G^1 H1	G^2 H1	T^3 H3	A^3 H2	T^4 H3	A^4 H2	T^5 H3	A^5 H2	G^6 H1	G^7 H1	G^8 H1	G^9 H1	T^{10} H3	A^{10} H2	T^{11} H3	A^{11} H2	G^{12} H1
σ^{ROE} (s^{-1})[b]	G^1 H1		3.1															
	G^2 H1	0.31		4.7	4.2													
	T^3 H3		0.03		3.0	3.1	3.2											
	A^3 H2		0.05	0.38		4.9	3.6											
	T^4 H3			0.31	0.02		2.7	4.2	3.9									
	A^4 H2			0.26	0.13	0.71		4.0	4.3									
	T^5 H3					0.05	0.07		2.7	3.6								
	A^5 H2					0.08	0.04	0.71		3.8								
	G^6 H1							0.13	0.09		3.3							
	G^7 H1									0.21		3.5						
	G^8 H1										0.15		3.4					
	G^9 H1											0.18		3.7	4.0			
	T^{10} H3												0.11		2.8	4.3	4.4	
	A^{10} H2												0.07	0.57		4.6	3.1	
	T^{11} H3													0.04	0.03		2.8	3.2
	A^{11} H2													0.04	0.31	0.57		4.1
	G^{12} H1															0.26	0.06	

[a] Distances over 5 Å are not shown in the upper triangular portion of the matrix.

[b] Blank cells in the lower triangular portion of the matrix indicate σ^{ROE} value of 0.

Supplementary Table S2. Calculated effective $R_{1\rho}$ relaxation rate

R_2^0 / $s^{-1\,a}$	${}^{eff}R_{1\rho}$ / s^{-1}						
	T^3	T^4	T^5	G^6	G^7	G^8	G^9
10	15.1	19.3	24.1	46.5	32.2	31.7	30.0
15	15.0	19.2	24.0	46.5	32.2	31.7	29.9
20	15.0	19.1	23.9	46.4	32.2	31.7	29.9
25	14.9	19.0	23.8	46.4	32.2	31.7	29.9
30	14.8	18.9	23.7	46.4	32.2	31.7	29.8
35	14.8	18.8	23.6	46.4	32.2	31.7	29.8
40	14.8	18.8	23.6	46.3	32.2	31.7	29.8
45	14.7	18.7	23.5	46.3	32.2	31.7	29.8
without σ^{ROE}	14.1[b]	17.9[b]	22.9[b]	45.7[b]	31.9[b]	31.4[b]	29.4[b]

[a] Auto relaxation rates used for ^1H resonances for which experimental R_2^0 values are unavilable.
[b] Effective $R_{1\rho}$ relaxation rate calculated without σ^{ROE}.
